# eXplainable Artificial Intelligence (XAI): A Systematic Review for Unveiling the Black Box Models and Their Relevance to Biomedical Imaging and Sensing

**DOI:** 10.3390/s25216649

**Published:** 2025-10-30

**Authors:** Nadeesha Hettikankanamage, Niusha Shafiabady, Fiona Chatteur, Robert M. X. Wu, Fareed Ud Din, Jianlong Zhou

**Affiliations:** 1 Design and Creative Technology, Torrens University Australia, 88 Wakefield St., Adelaide, SA 5000, Australia; 2 Faculty of Science and Technology (Sydney Campus), Charles Darwin University, 815 George Street, Sydney, NSW 2000, Australia; 3 Discipline of Information Technology, Peter Faber Business School, Australian Catholic University, Sydney, NSW 2060, Australia; 4 Design and Creative Technology, Torrens University Australia, 46-52 Mountain St., Sydney, NSW 2007, Australia; 5 Faculty of Engineering and Information Technology, University of Technology Sydney, 15 Broadway, Sydney, NSW 2007, Australia; 6 School of Science and Technology, University of New England, Armidale, NSW 2350, Australia; 7 Data Science Institute, University of Technology Sydney, 15 Broadway, Sydney, NSW 2007, Australia

**Keywords:** eXplainable artificial intelligence, machine learning, quantitative prediction, PRISMA, shapley additive eXPlanations (SHAP), model-agnostic explanations (LIME), partial dependence plots (PDPs), permutation feature index (PFI)

## Abstract

Artificial Intelligence (AI) has achieved immense progress in recent years across a wide array of application domains, with biomedical imaging and sensing emerging as particularly impactful areas. However, the integration of AI in safety-critical fields, particularly biomedical domains, continues to face a major challenge of explainability arising from the opacity of complex prediction models. Overcoming this obstacle falls within the realm of eXplainable Artificial Intelligence (XAI), which is widely acknowledged as an essential aspect for successfully implementing and accepting AI techniques in practical applications to ensure transparency, fairness, and accountability in the decision-making processes and mitigate potential biases. This article provides a systematic cross-domain review of XAI techniques applied to quantitative prediction tasks, with a focus on their methodological relevance and potential adaptation to biomedical imaging and sensing. To achieve this, following PRISMA guidelines, we conducted an analysis of 44 Q1 journal articles that utilised XAI techniques for prediction applications across different fields where quantitative databases were used, and their contributions to explaining the predictions were studied. As a result, 13 XAI techniques were identified for prediction tasks. Shapley Additive eXPlanations (SHAP) was identified in 35 out of 44 articles, reflecting its frequent computational use for feature-importance ranking and model interpretation. Local Interpretable Model-Agnostic Explanations (LIME), Partial Dependence Plots (PDPs), and Permutation Feature Index (PFI) ranked second, third, and fourth in popularity, respectively. The study also recognises theoretical limitations of SHAP and related model-agnostic methods, such as their additive and causal assumptions, which are particularly critical in heterogeneous biomedical data. Furthermore, a synthesis of the reviewed studies reveals that while many provide computational evaluation of explanations, none include structured human–subject usability validation, underscoring an important research gap for clinical translation. Overall, this study offers an integrated understanding of quantitative XAI techniques, identifies methodological and usability gaps for biomedical adaptation, and provides guidance for future research aimed at safe and interpretable AI deployment in biomedical imaging and sensing.

## 1. Introduction

Artificial Intelligence (AI) has become increasingly established in prediction applications across various fields, such as healthcare [1], engineering [2], energy [3], finance [4], and business [5]. However, AI systems have become more complex, and their decision-making process often becomes less transparent and hard to understand [6]. This situation hinders practitioners’ understanding, leading to concerns about bias, untrustworthiness, and discrimination. Therefore, an interpretation of the AI models’ outcomes and work is required to enhance the decision-making process in various applications.

Explainable Artificial Intelligence (XAI) has emerged as an important research area that refers to the features that explain how the AI methods constructed their prediction outcomes [7,8]. The goal of XAI is to create AI models and algorithms that can be easily understood and interpreted by humans, enabling users to trust and rely on AI systems for critical decision-making [8]. Traditionally, many AI methods have been known as black-box models, which means that their decision-making processes were opaque and complex for humans to understand [9]. This lack of transparency made it challenging for users to know how and why the AI systems were making certain decisions. XAI aims to solve these problems by creating AI models that can provide clear interpretable explanations for their actions [9].

Recent advances in XAI have especially focused on medical imaging and sensing applications, given their critical importance in diagnostics, monitoring, patient safety, and regulatory oversight. For example, examining the current landscape of XAI in medical imaging, including visual, textual, and example-based explanations [10] and self-explainable AI for medical image analysis [11] to highlight models that incorporate explanations inherently rather than post hoc. The potential of XAI techniques demonstrate both the urgency and rapid evolution of XAI techniques in biomedical imaging fields.

XAI has gained significant attention in recent years due to the black-box nature of AI models and the need for explanations behind how these models made their predictions [8]. Numerous survey papers have been published to provide a comprehensive overview of the current state of XAI, especially during the hype cycle during 2017 to 2023. In order to highly the gap/need for XAI techniques and their relevance to biomedical imaging and sensing, this paper also unified previous efforts and presented a complete taxonomy of XAI techniques [10]. Some of them focused on a specific domain, such as healthcare and automation [12], exploring the application of XAI on various prediction tasks. For example, Chaddad et al. [13] conducted a survey to explore XAI techniques for medical imaging applications by emphasising algorithms used to improve interpretability and address challenges of XAI methods in the medical domain. The study also provided instructions for developing better interpretations of deep learning models in medical image and text analysis. Another paper investigated the utilisation of XAI techniques for skin cancer detection [14]. XAI methods have been applied in identifying pandemic scenarios; for example, the systematic review conducted by Giuste et al. [15] focused on examining XAI application in combatting pandemics. This study evaluated the XAI performances for enhancing the value of AI-based decision support systems. This paper also presented the traditional and modern advanced XAI techniques and provided best practice guidelines for AI users. The automotive industry is one of the specific domains that has witnessed of application of AI, such as the review conducted by Omeiza et al. [16] to observe explanations by highlighting the transparency, accountability, and trust in automation vehicle systems. Several additional survey articles have been carried out with the objective of presenting categorisations of XAI techniques such as Minh et al. [17] to conduct a survey to investigate XAI techniques and categorise them into pre-modelling and post-modelling. This study paid attention to systematically discussing the XAI techniques challenges, evaluation methods, security, and policy.

For the purposes of this review, ‘quantitative data’ refers to structured numeric data and numerical features derived from sensing or imaging sources (for example: numerical measurements extracted from medical images, time-series signals from wearable sensors, tabular clinical variables, and derived biomarkers). We therefore excluded studies whose primary data modality was raw unstructured image or text data without accompanying numeric features, or those where explanation focused on visual saliency alone (e.g., Grad-CAM heatmaps used without corresponding numeric feature explanations). The objective is to synthesise XAI methods most directly applicable to numerical prediction tasks—i.e., tasks that produce numeric or class predictions based on quantitative inputs, as these settings are highly relevant for sensor outputs, derived imaging biomarkers and many clinical decision-support scenarios.

It is worth noting that the majority of XAI review articles focus on method summaries, categorisations, or specific domains and give little attention to exploring the use of XAI techniques in predictions across various disciplines. Due to the lack of contribution of XAI surveys to the predictions, this study is motivated to explore the lacking context of XAI application and their relevance to biomedical imaging and sensing. By explicitly focusing on predictions where the quantitative data was utilised, this survey aims to identify and evaluate the various XAI methods employed in a wide array of domains. While this review draws from quantitative-prediction applications across many domains, we emphasise the results and lessons in the context of biomedical imaging and sensing, as these domains best illustrate real-world stakes and the need for interpretability, fairness, and accountability, especially when predictive models inform diagnosis, treatment, or patient-monitoring. The review of these applications serves as an essential guide in providing valuable insights to stakeholders enabling them to make informed decisions when choosing appropriate XAI techniques. By understanding the benefits, challenges, and trade-offs of different XAI techniques, developers can effectively enhance the explainability and interpretability of these techniques, thus fostering the broader implementation of AI systems in real-world scenarios.

Despite the extensive body of research on XAI, including review studies, this review narrows the focus by specifically exploring Q1 journal articles that present the applications XAI techniques for quantitative predictions in various domains. The choice of focusing on quantitative studies is that predictions on quantitative data play a significant role in many practical disciplines, such as healthcare, engineering, energy, finance, and business. Interpretation of the prediction outcomes in these sectors is therefore essential for decision-making, resource allocation, and risk assessment. The contribution of this paper includes the following:

- Identify and categorise XAI techniques applied for quantitative prediction tasks across diverse domains, and their relevance to biomedical imaging and sensing.
- Highlight the advancements, challenges, and benefits of the XAI techniques applied for numerical prediction tasks.
- Identify the gaps and provide future research direction in applying XAI techniques for predictions.

The structure of this paper is outlined as follows. Section 2 describes the strategies applied for the research and selection of the data. Section 3 illustrates the XAI techniques and the corresponding prediction applications in various domains. Section 4 discusses the review summary and challenges in existing applications and future direction. Section 5 presents the conclusion of the study.

## 2. Research Methodology

This study adopted the ‘Preferred Reporting Items for Systematic Reviews and Meta-Analysis’ (PRISMA) [18] protocol as a guide in the development of the methodology. Creating a well-defined research question (RQ) is the first step in conducting a systematic review, encapsulating the main objective of the study [19]. In line with this, the following RQ has been formulated; What XAI techniques have contributed to prediction applications using quantitative data?

### 2.1. Search Strategy

To conduct this systematic review, we devised a search strategy to locate and identify the relevant literature. This search strategy was tailored to two databases, Science Direct and the Institute of Electrical and Electronics Engineers (IEEE), covering XAI techniques in prediction applications in all fields. The following keywords and combinations were applied in the search string: “explainable AI” OR “interpretable AI” OR “explainable artificial intelligence” OR “interpretable artificial intelligence” OR “explainable machine learning” OR “interpretable machine learning” (see Table 1). All searches spanned the databases from 2017 to 2023.

The review window was restricted to the period from 2017 to 2023. This period was deliberately chosen because it captures the formative years and peak of the first XAI hype cycle: following seminal contributions (e.g., LIME in 2016 and SHAP in 2017), the years 2017–2023 saw rapid proliferation and consolidation of model-agnostic explainability techniques across domains. This window selection strengthens the focus to capture the ‘first maturity wave’ and the hype-cycle phase of XAI, during which foundational methods such as SHAP and LIME transitioned from theoretical development to applied use in diverse quantitative domains, including the initial uptake within biomedical imaging and sensing. Although a few post-2023 biomedical-specific reviews have appeared, these serve mainly to confirm that XAI’s conceptual evolution identified in this study continues into more domain-specific applications. Focusing on this window allowed a comprehensive review of foundational methods that shaped the current state of XAI and continue to underpin later biomedical applications.

We restricted our formal database searches to ScienceDirect and IEEE Xplore because our objective was to identify peer-reviewed, full-length journal articles with detailed methodological reporting, and both databases index a broad set of Q1 journals across applied sciences. This design allowed cross-domain comparison of XAI implementations across biomedical, engineering, energy, and economic contexts, which was central to the purpose of this review. We acknowledge that dedicated biomedical databases such as PubMed/PMC or specialty collections within Web of Science may include additional domain-specific XAI studies. However, during our preliminary scoping, we performed targeted scans in Google Scholar to identify potentially relevant biomedical XAI studies and recent systematic reviews published after 2023. As Google Scholar aggregates content from both PubMed and ScienceDirect, many PubMed-indexed works that are hosted on ScienceDirect were indirectly captured during this stage, thereby minimising duplication and missed coverage of relevant biomedical papers. This overlap meant that many PubMed-indexed articles with full-text access via ScienceDirect were already captured through our selected databases, indirectly broadening biomedical coverage while avoiding duplication.

The scoping exercise revealed a limited number of newer biomedical XAI reviews (for example, deep-learning-based explainability [10] and self-XAI for medical imaging [11]), but these primarily focused on image-saliency or qualitative explanation methods and lacked quantitative feature-level analyses required for our inclusion criteria. Consequently, they were not included in the formal synthesis. We nevertheless acknowledge the absence of formal PubMed searching as a limitation (see Section 4.1). Future studies focused exclusively on the biomedical domain should extend database coverage to include PubMed and other specialist medical indexes to ensure comprehensive retrieval of the emerging literature. This review was conducted according to the PRISMA 2020 guidelines [18]. The review protocol was not prospectively registered in PROSPERO or other registries. However, the PRISMA 2020 checklist is presented in as Supplementary Material.

### 2.2. Selection Criteria and Quality Assessment

**Figure 1:**
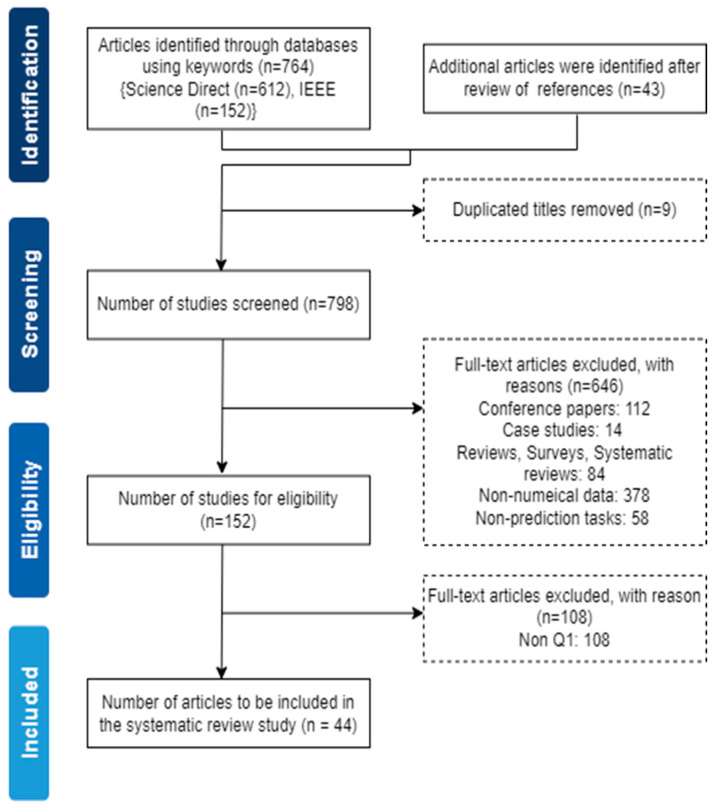
PRISMA 2020 systematic review filtration protocol [18].

The selection criteria were based on the PRISMA protocol [18] (see Figure 1). The search mainly focused on mapping the existing literature on applying explainable artificial intelligence (XAI) techniques’ contribution to predictions in all fields and, in particular, their relevance to biomedical imaging and sensing. This study started with 764 search results in the selected journal databases using the Boolean search string (see Table 1), and 43 studies were identified after reviewing the references. After removing the 9 duplicated publications across the journal databases, 798 articles were screened with the titles and abstracts of the full-text original articles. Following that, we removed 646 articles, comprising conference papers, case studies, the literature reviews, surveys, systematic reviews, and XAI techniques implemented on non-prediction problems. We were left with 152 full-text articles eligible to be taken to the next step. For this process, 108 articles were removed from non-Q1 journals: the quartile (Q) of the journal was acquired from SJR-Scientific Journal Ranking [19]. Consequently, we included 44 full-text original articles sourced exclusively from Q1 journals. The choice of selecting Q1 journal articles for the survey is that the Q1 journals are known for their rigorous peer-review process and high-quality standards, ensuring that the relevant articles have been undertaken through in-depth examination by experts in the field. In addition, the screening strategy recognised that seminal methodological papers (for example, the original SHAP exposition by Lundberg and Lee, published in NeurIPS 2017 [20]) are highly relevant to interpreting current practice although they appear in conference proceedings rather than Q1 journals. Where such seminal methods were not themselves eligible for formal inclusion (because they fell outside the Q1-journal inclusion criterion), they were nevertheless discussed and cited to provide methodological context. To maintain the quality of the process, the search criteria were checked thoroughly with the help of an independent reviewer/librarian. To minimise bias in study selection and data extraction, an independent reviewer with expertise in systematic searching (a research librarian) was involved in the screening process. Disagreements between the lead author and the independent reviewer were resolved through discussion until consensus was reached. A list of full-text articles excluded at the eligibility stage with reasons is available from the corresponding author upon reasonable request.

### 2.3. Data Extraction and Analysis

At this stage, for data extraction and analysis of the 44 articles, we separated them into constituent parts based on addressing the specific attributes of the research question (What XAI techniques have contributed to prediction applications using quantitative data?). These attributes are as follows:

- The RQ specifically examines the XAI techniques, which aim to provide explanations for the prediction outcomes generated by AI models.
- The RQ concerns predictions involving quantitative data.
- The RQ seeks to identify the contribution of XAI methods to the prediction problems in various domains, how these techniques have interpreted the outcomes, and potentially uncover any limitations associated with their use.

## 3. Results

Due to the recent introduction of XAI, it is understandable that the first Q1 journal article dedicated to XAI contribution to prediction tasks emerged in 2017. There were no dedicated studies in the XAI application for predictions in Q1 journals in 2018. The application of XAI techniques for prediction tasks has increased exponentially between 2019 to 2023.

**Figure 2:**
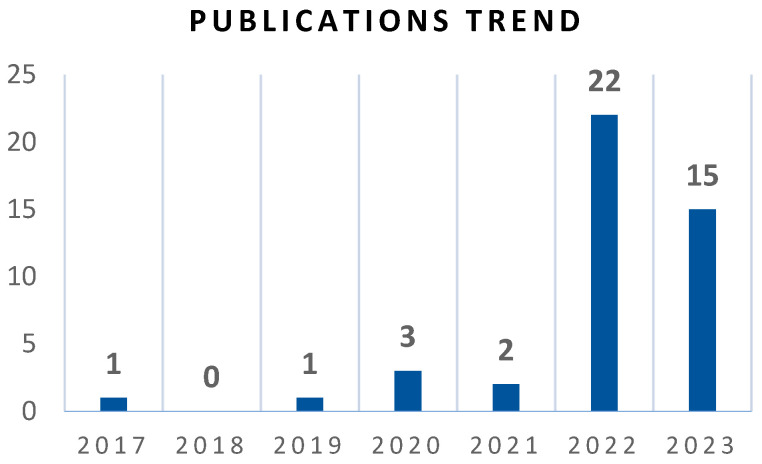
XAI articles from 2017 to 2023.

A comprehensive collection of 44 Q1 journal articles has been analysed, highlighting the adaptation of XAI techniques across various fields (see Figure 2), as evidenced by several studies. Among the 44 studies analysed, biomedical imaging and sensing represent a minority but critically important subset. Nevertheless, several recent works, such as [10,11], are almost exclusively focused on medical imaging, which suggests that the next wave of publications are increasingly biomedical in orientation. Thus, our findings are highly relevant as they anticipate and overlap with these emerging studies. Among other domain, we categorise studies from the selected pool of papers, there were 10 articles in the civil engineering domain, which utilised XAI techniques to interpret the structural analysis. Eight of the 44 articles studied used XAI techniques in healthcare. The field of energy used explainability in 6 out of the 44 journal articles. In finance, XAI techniques have been utilised and reported by 5 out of the 44 articles. Environmental studies have recognised the potential of applying XAI methods in 4 studies. Additionally, two studies in chemical engineering and economics have employed interpretable techniques to enhance the decision-making process. Furthermore, explainable AI methods have been utilised in other domains, including aeronautics, aviation, genomics, mechanical engineering, mining, and sports. Lastly, XAI also made an impact in miscellaneous studies in which we were unable to identify the related industry. Table A1 (in Appendix A) summarises the included 44 studies that have implemented XAI techniques for prediction problems. These articles underline the influence of explainable AI methods across various sectors, fostering interpretability and informed decision-making. The application of each XAI technique will be broadly discussed in the later section.

**Figure 3:**
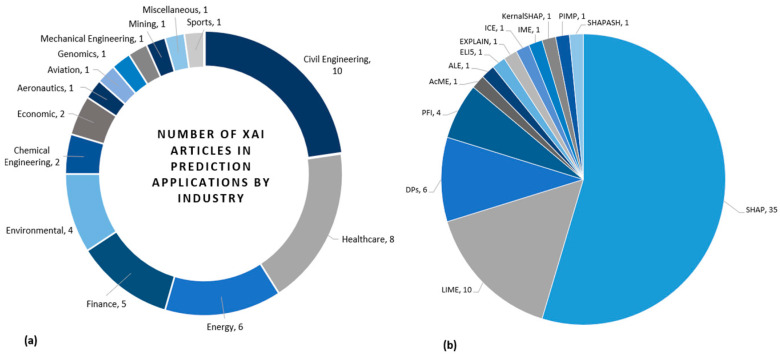
(**a**) Number of XAI articles in different industries. (**b**) Employed XAI techniques/models.

**Figure 4:**
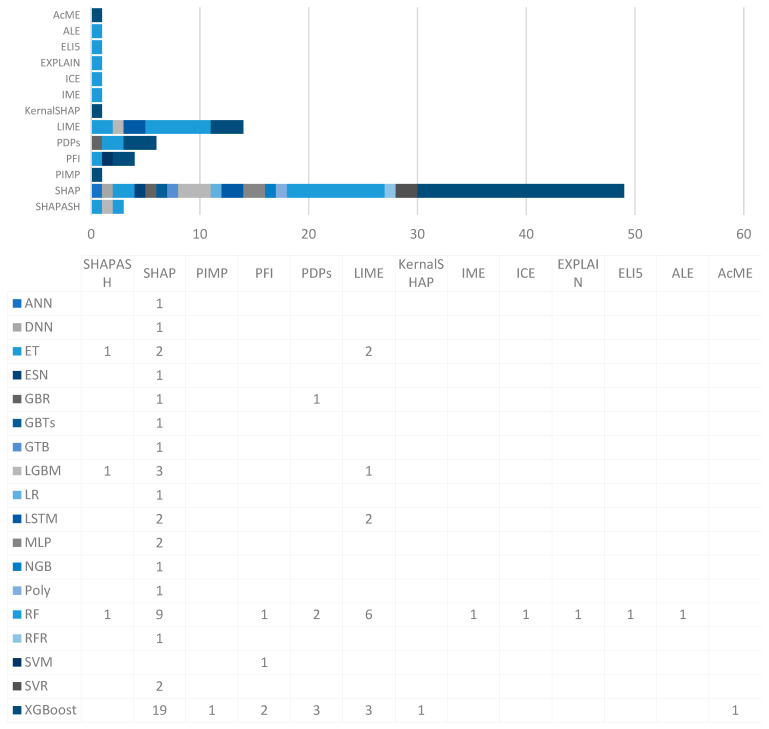
Illustration of the distribution of prediction models in conjunction with XAI techniques.

According to the result, the most popular XAI techniques are Shapley Additive eXPlanations (SHAP), Local Interpretable Model-Agnostic Explanations (LIME), Partial Dependence Plots (PDPs), and Permutation Feature Index (PFI), utilising 35, 10, 6, and 4 articles, respectively (see Figure 3). Some articles used more than one technique. Other identified models, including Accumulated Local Effects (AcME), Additive Feature Attribution (ALE), Explain Like Im 5 (ELI5), EXPLAIN, Individual Conditional Expectation (ICE), IME, KernalSHAP, Permutation Importance (PIMP), and SHAPASH used by one study each technique. Figure 4 illustrates the distribution of prediction models in conjunction with each XAI technique. The more frequently used machine learning prediction models are Extreme Gradient Boosting (XGBoost), Random Forest (RF), Extra Tree (ET), Echo State Network (ESN), Long Short-Term Memory (LSTM), Gradient Boosting Regression (GBR), Light Gradient Boosted Machine (LGBM), Multi-Layered Perceptron (MLP), Deep Neural Network (DNN), Gradient Boosted Tree (GBTs), Gradient Tree Boosting (GTB), Logistic Regression (LR), Natural Gradient Boosting (NGB), Polynomial (Poly), Random Forest Regression (RFR), Support Vector Machine (SVM), and Support Vector Regression (SVR).

In the upcoming sections, we provide an analysis of each XAI technique identified for prediction applications. This will involve a thorough examination and explanation of the various XAI methods, ensuring a comprehensive understanding of them. The XAI techniques are organised based on their popularity.

### 3.1. Shapley Additive Explanations (SHAP)

Shapley Additive explanations (SHAP) is a local and global interpretation technique that aims to provide a better understanding of machine learning models by calculating feature importance values for individual predictions [20]. This method is inspired by game theory, especially the concept of Shapley values, which is used to calculate the contribution of each player in a collaborative game [21]. The approach used the prediction as the “playout” and each feature value as a “player” in the game. The goal is to ensure fair distribution of payouts to players. Given a trained model f and a generic data point x, represented by an n-dimensional feature vector (x∈R), SHAP computes, for each feature that represents the contribution of the feature to the prediction $f\left(x\right).$ SHAP attribute $\emptyset_{i}$ to each predictor (feature), and the sum of these effects, $g\left(z^{\prime }\right),$ approximates the output $f\left(x\right)$ of the original model. This can be formulated as follows:

(1) $$g\left(z^{\prime }\right)=\emptyset_{0}+\sum_{i=1}^{N}\emptyset_{i}z_{i}^{\prime },\emptyset_{i}\in R$$

where N is the number of input features in x, the instance vector,

g is the explanation model,

$z^{i}$ is the coalition vector such that $z^{i}\in {\left(0,1\right)}^{N}$,

$\emptyset_{i}$ is the decomposition factor.

Given a trained model f and a generic data point x, represented by an n-dimensional feature vector (x∈R), SHAP computes, for each feature that represents the contribution of the feature to the prediction $f\left(x\right).$ SHAP attribute $\emptyset_{i}$ to each predictor (feature), and the sum of these effects, $g\left(z^{\prime }\right),$ approximates the output $f\left(x\right)$ of the original model. In Equation (1), the SHAP values $\emptyset_{i}$ are defined to satisfy additive attribution $\emptyset_{0}+\sum_{i=1}^{N}\emptyset_{i}$ where $\emptyset_{0}$ is the expected base value. Computation of SHAP values relies upon a background distribution for marginalisation (commonly the training set distribution), and for correlated features the marginal or conditional expectation choices change attributions [22].

SHAP assumes an additive explanation model and the decomposition relies on the definition of the background distribution used to marginalise features; choices of background and handling of correlated features materially affect attributions [22]. SHAP computes additive feature attributions that sum to the model output; its interaction values provide a decomposition of pairwise interaction contributions under the additive Shapley framework. While SHAP is widely used because it yields consistent, locally exact additive attributions, critical limitations have been identified in the literature, e.g., potential sensitivity to feature correlation [23], questionable causal interpretation when features are not independent [24], and the fact that Shapley axioms (developed for homogeneous payoff distributions) do not automatically ensure domain-appropriate explanations in heterogeneous settings such as biomedicine [24]. In general, SHAP provides two distinct advantages, comprising global and local explainability of AI methods. Unlike other significant features in AI models, SHAP can evaluate the positive and negative influence of each input characteristic [1].

**Figure 5:**
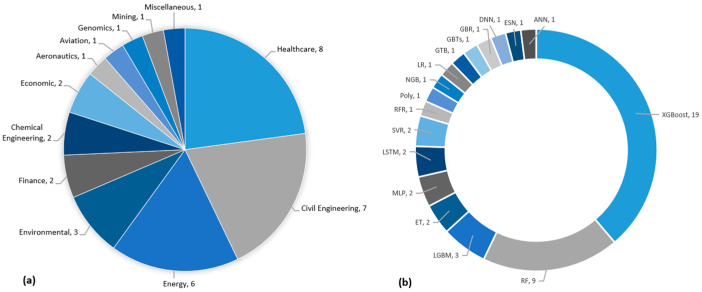
(**a**) SHAP utilisation by industry. (**b**) AI methods used in SHAP studies.

SHAP has been a widely employed technique having been used in 35 out of the 44 articles included in this study (see Figure 5). It is a popular XAI tool in various fields for prediction applications, and the most prominent areas are healthcare, civil engineering, and energy. In healthcare, SHAP has been employed by eight studies, demonstrating its potential for early medical diagnoses [1,25,26], future treatment targets, prediction of post-operative mortality [27], informing clinical decisions [28], personalising medicine applications, and in understanding the logic governing the prediction outcomes [29]. Figure 5b illustrates the machine learning prediction models that have been explored and implemented for various prediction tasks in conjunction with SHAP. Among these AI models, XGBoost stands outs, with 19 studies out of 35 showcasing its combination with SHAP for prediction interpretability. RF followed by 8 articles out of 35, highlighting its compatibility with SHAP in various domains. ET has been employed by two articles, where SHAP-based analyses demonstrated computational consistency of the predictive outcomes. Additionally, other models such as RFR, Poly, NGM, MLP, LSTM, LR, LGBM, GTB, GBTs, GBR, and DNN have each been featured in one study, respectively, highlighting their successful integration with SHAP for prediction applications. These findings emphasise the diverse range of machine learning and deep learning models that have been computationally integrated with SHAP to generate post hoc explanations and model-interpretation outputs.

SHAP has been widely used in civil engineering, as evidenced by seven studies to generate post hoc attributions, as it quantifies the contribution of each feature in a sample to machine learning predictions. These seven studies highlight the computational effectiveness of SHAP in various purposes from an algorithmic standpoint, such as for liquefaction assessment of soils [30], the probabilistic buckling stress prediction of steel shear panel dampers [31], predicting demands for designing and assessing structures under seismic loads [32], and predicting the shear capacity of FRP-RC (fibre-reinforced polymer-reinforced concrete) beams [33]. Additionally, SHAP has been employed for identifying the failure mode of flat slabs [34] and the wind pressure of a low-rise building [35], and accelerating the development of one-part alkali-activated materials with the desired properties [36]. However, none of the reviewed works included structured user or clinician usability evaluations to confirm whether these computational explanations translated into improved human understanding or decision-making. Therefore, the term effectiveness here refers exclusively to computational and analytical performance, not validated user-centred interpretability.

The predominance of model-agnostic feature importance techniques (e.g., SHAP, PDP) in our dataset finds echoes in recent imaging-focused reviews such as Explainable AI for medical imaging systems using deep learning [10,11], where SHAP and Grad-CAM are again among the most used. This reinforces that these methods are central to computational employability for both, in general, quantitative prediction and more specifically in medical imaging contexts in the midst of all other domain applications. Nonetheless, their centrality reflects prevalence in computational analyses rather than evidence from usability or clinical validation studies. The reviewed corpus indicates that, despite their frequent use, systematic user evaluations confirming their interpretability benefits remain absent.

In the energy industry, SHAP has been extensively employed to present the computational validity evaluate and analyse prediction outcomes obtained by machine learning prediction models. In the selected pool of extracted work, six studies underlined the efficacy of SHAP in fulfilling diverse objectives in a computational environment, such as the estimation of reservoir temperature of geothermal systems [37], analyse of secondary control power [38], power factors of diamond-like thermoelectric materials [39], solid yields and higher value of torrefied biomass [40], building load prediction [41], and photovoltaic power generation [7]. By integrating SHAP, these studies provided insights into the underlying factors and variables that influence the prediction results in each respective study. The interpretations facilitate informed decisions and advancements in the energy field. However, these improvements were evaluated computationally rather than through user-centred or operational validation, a limitation that similarly applies to most studies across other domains, including biomedical imaging and sensing.

In the environmental field, SHAP has been employed in three articles to gain insights into various environmental phenomena. A recent study conducted by El Bilali et al. [42], integrated SHAP in their proposed work to interpret the machine learning models in predicting daily pan evaporation. Another study applied SHAP to interpret transition water quality from eutrophic to hypereutrophic states [43] and predict biological stream conditions. Maloney et al. [44] also focused on predicting biological stream conditions. Both studies highlighted that the SHAP had provided insights into the relative importance of various environmental features in predictions.

In finance, two articles employed SHAP to enhance their model interpretation. An article authored by Ghosh et al. [45] identified the significant impact of media chatter on stock market prices during the COVID-19 pandemic. By incorporating systematic media chatter indices, authors aimed to monitor various orthodox technical indicators and macroeconomic variables. To better understand the model’s outcomes, they employed SHAP to generate explanations for individual predictions. Weng et al. [46] highlighted the importance of knowing the relationship between financial pressure and stock price volatility in the healthcare domain. With the implementation of SHAP, they could analyse the specific financial indicators that had influenced the instability of healthcare stocks. These applications highlight SHAP’s recurring use as a computational tool for post hoc model interpretation, yielding analytical insights into feature importance and prediction drivers within the finance domain.

The chemical engineering field has discovered the value of interpretability in complex phenomena. Two notable studies were employed SHAP to understand the complexity behind prediction models. These studies used SHAP to interpret the quantitative relationship between each physicochemical property in carbon dioxide adoption on porous carbon [47] and the absorption wavelength of azo dyes [48]. In both studies, SHAP was used to provide feature-level attributions that the authors interpreted to gain insights into chemical relationships; however, these studies did not perform formal human-usability testing to confirm whether these attributions improve human understanding or decision outcomes.

In economics, SHAP has been employed in two articles. A study conducted by Park and Yang, [49] employed SHAP in order to provide an interpretation of the prediction model that derives economic patterns of growth and crisis. Another study conducted by Rico-Juan and Taltavull de La Paz, [50], utilised SHAP in their approach to provide the explainability of housing-price prediction outcomes. These two articles summarised that interpreting AI models would add information on the unobservable relationships between variables and accelerate informed decisions.

SHAP has also been used in other domains such as aeronautics, aviation, genomics, and mining studies, with each field having one study dedicated to its application on prediction problems. A study in aeronautics by Baptista et al. [51] emphasised the significance of making correct decisions on prognostic tasks which contain several hundred run-to-failure trajectories in jet engines. Through the utilisation of SHAP, the study was able to track the metrics associated with prediction outcomes. In the field of aviation, a study conducted by Midtfjord et al. [52] employed SHAP to interpret the outcomes of predicting runaway conditions. The study aimed to improve airport operations’ decision support systems, contributing to safer and more economical operations, which aligns with overall performance efficiency [53]. The genomics study conducted by He et al. [54] aimed to accelerate the evaluation of the martensitic transformation peak temperature of high entropy memory alloys using ML models. The interpretation of the ML models performed by SHAP demonstrates the crucial role of Allred Rochow electronegative in predicting peak temperature. In the mining field, one notable study [55] has used SHAP to interpret the predictions of ash content, highlighting the nine most important elements that significantly influence the outcomes.

In addition, one notable study conducted by Dandolo et al. [56] used XAI techniques in the context of human-in-the-loop, such as decision support systems to interpret the feature importance of data. As the specific industry of this study was somewhat unclear, this work has been categorised under miscellaneous. This study has utilised SHAP as a benchmark to provide a comparative evaluation alongside their work. The integration of XAI methods into decision support systems is crucial for facilitating corrective actions in the decision-making process.

### 3.2. Local Interpretable Model-Agnostic Explanations (LIME)

Local Interpretable Model-agnostic Explanations (LIME) was first introduced by Ribeiro et al. [57]. LIME has gained popularity as one of the most effective interpretable techniques for black-box methods. This approach provides a straightforward yet effective approach to generate interpretations for individual prediction scores produced by any classifier. It creates simulated data points around a given input instance for which the classifier produced a prediction. These simulated instances are then used to make new predictions using the same classifier, with their proximity to the original instance considered. This local model can be interpreted to gain insights into the initial black-box model’s decision-making process. However, despite its effectiveness and simplicity, it has its drawbacks. LIME’s explanations are local approximations and depend strongly on the neighbourhood sampling strategy and kernel width; these parameters can produce unstable explanations across runs and may fail to capture global model behaviour. Empirical and theoretical critiques highlight cases where LIME can omit significant features or produce misleading local models when features are highly correlated or non-linear globally [23]. A theoretical analysis of LIME was conducted by Garreau and Luxburg, [58], which verified the importance and value of LIME in producing meaningful interpretations. However, the analysis also revealed that improper selection of parameters could lead to it missing significant features. The LIME can be formulated as below,

(2) $$y\left(x\right)=argminL\left(f,g,\pi_{x}\right)+\Omega (g)$$

where L is used to measure the interpretable model,

argmin is the ‘argument of the minimum’, which is used to search the model g

g is the prediction of the original complex model f,

f is the original complex model.

$\pi_{x}$ represents the proximity of the sampled instances to the instance x,

Ω(g) is the complexity of model g.

LIME constructs an interpretable local model g by sampling perturbed instances z in the neighbourhood of x and weighting them via a kernel π_x(z). The choice of the perturbation distribution and $\pi_{x}$ critically influences interpretability results; see Ribeiro et al. (2016) [57] and Garreau and von Luxburg [58] for formal analysis.

**Figure 6:**
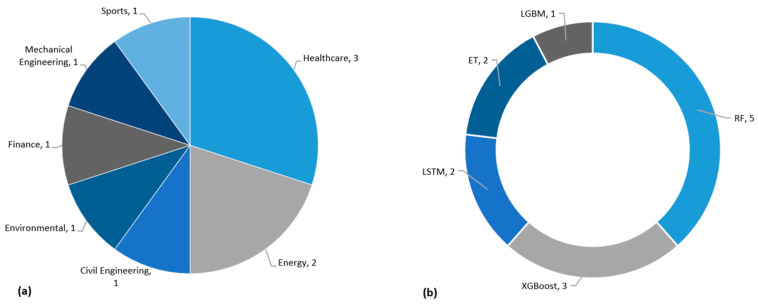
(**a**) LIME utilisation by industry. (**b**) AI methods used in LIME studies.

This systematic review has revealed that LIME had been utilised in 10 out of the 44 studies. LIME has gained significant attention as an XAI technique for prediction applications in various domains, including healthcare, civil engineering, energy, environmental, finance, mechanical engineering, and sports, as depicted in Figure 6a. In healthcare, three studies explored the application of LIME for predictive analytics. These three articles have also been discussed under SHAP. In energy, LIME has been used in two studies [7,41]. Similarly, LIME has been implemented in civil engineering, finance, mechanical engineering, and sports, with one article each [1,26,28,42]. In 4 out of 10 studies, LIME was employed instead of SHAP to interpret the prediction outcomes. These four articles have been used for the interpretation of the following prediction applications; stock market prediction [59], heat demand forecasting for control strategies [60], gameplay prediction of the national basketball association (NBA) [61] and evaluating data-driven building energy performance [62].

With the integration of LIME, various prediction models have been employed for the aforementioned machine learning prediction applications (see Figure 6). Among these methods, RF in conjunction with LIME was utilised in 5 scenarios, showcasing its versatility and robustness in handling complex datasets. XGBoost is another prediction model, which has been employed in three instances integrated with LIME, highlighting its effectiveness in prediction problems. LGBM, known for its efficacy and high performance, was employed in one study in conjunction with LIME. LSTM has been used in two instances, showcasing its suitability for temporal prediction problems. Lastly, ET has been employed in one scenario, emphasising its prediction performance integrated with LIME. The interpretation of these models’ prediction performance with LIME provides valuable insights into the decision-making process [59].

### 3.3. Partial Dependence Plots (PDPs)

Friedman [63] introduced PDPs, a visualisation tool that facilitates the interpretation of any opaque prediction model by demonstrating the influence of specific features or subsets of features on the model’s predictions. PDPs display how a particular set of features impacts the average predicted value by eliminating the effects of the remaining features (its complement feature set). PDPs are usually simplistic and do not account for all possible feature interactions, resulting in an inaccurate approximation of the true functional relationships between dependent and independent variables. Nonetheless, they can still offer valuable insights, especially in cases where most interactions are of low order, aiding in the interaction of black-box models. PDPs can provide visualisations for single and multi-class problems and exhibit feature interactions. The mathematical formulation of PDPs can be expressed as follows [64].

(3) $${\hat{f}}_{x_{s}}\left(x_{s}\right)=\frac{1}{n}\sum_{i=1}^{n}{\hat{f}}_{x_{s}}\left(x_{s},x_{c}^{i}\right)$$

where $x_{s}$ illustrates the features inspected through PDP,

$x_{c}$ expresses the remaining features, and

$\hat{f}$ denotes the utilised machine learning model.

This systematic review of XAI techniques in Q1 journal articles revealed that PDPs were implemented in 6 out of 44 prediction applications. Among these six studies, four studies have already been discussed under the SHAP technique, which has implemented XAI techniques for prediction analysis [25,44,46,56]. The remaining studies employed PDPs to determine the importance of features in the prediction results of ML models. Among them, Qian et al. [64] aimed at financial distress prediction and employed PDPs to identify the average marginal effect of features influenced on prediction outcomes. Another study conducted by Zhao et al. [65] implemented PDPs to evaluate the prediction outcomes of hydrogen production through supercritical water gasification of biomass. The use of PDPs in these studies highlighted their versatility as an XAI technique not only in helping to understanding the marginal effect of individual features but also in contributing to the interpretation of the outcomes.

### 3.4. Permutation Feature Importance (PFI)

The PFI is a model-agnostic method that provides global interpretability by inspecting the model score after randomly shuffling a single feature [66]. The increase or decrease in the model score describes the relationship between the prediction and the permuted feature. PFI replaces each feature p times with other features from randomly selected instances from the dataset. Firstly, the score of the classifier is computed. Then each feature is shuffled, generating a perturbed version of the test dataset, D-set. The recalculated model score corresponds to the score obtained when using the permuted dataset. Lastly, the importance of each feature is determined by calculating the difference between the initial model score and the average of the model scores obtained with the permuted data, which is repeated p times. The higher the decrease in the model’s score, the more relevant the feature is. The fundamental idea of PFI is that if a particular input variable $\left(x_{i}\right)$ holds great influence over the outcome, the forecast accuracy will decrease by randomly shuffling $\left(x_{i}\right)$ during which the order of other variables unchanged [66]. The PFI can be defined as below, where ${MAE}_{perm}$ and ${MAE}_{orig}$ are the mean absolute error before and after the randomly adjusted $x_{i}$ sequence [67]. As the PFI value is closer to zero, its impact on the output diminishes, while high values of PFI imply a more significant influence on the output [67].

(4) $$PFI={MAE}_{perm}-{MAE}_{orig}$$

This systematic review of the various studies of candidate XAI techniques has revealed that PDPs were implemented in 4 out of the 44 machine learning prediction applications. Among these four studies, two studies have already been discussed under the SHAP technique, which implemented XAI techniques for prediction analysis [25,28]. The remaining two studies employed PFI in the field of civil engineering. Peng and Unluer, [66] used the PFI technique to evaluate the influence weight of each input parameter on the prediction outcomes. The analysis thereby demonstrated a speculative basis for improving the mechanical properties of geopolymer concrete. Another study conducted by Almustafa and Nehdi, [67] utilised PFI in predicting the structural response of reinforced concrete slabs exposed to blast loading.

### 3.5. Accelerated Model-Agnostic Explanations (AcME)

The Accelerated Model-agnostic Explanations (AcME) method was proposed by Dandolo et al. [56] for machine learning models’ explainability. The AcME aims to analyse the contribution of each input feature at both global and local scales. To establish the importance scores, AcME uses perturbations of the data on the quantiles of the observed distribution of each attribute. These perturbations are implemented with a particular reference point in the input space, also known as the baseline vector (denoted as $x^{b}$). The AcME can be used in conjunction with regression or classification models based on tabular data. The proposed model was applied to the context of human-in-the-loop ML applications where users need to take corrective actions immediately (e.g., decision support systems for fraud detection). In order to evaluate the proposed work, this study has used previously discussed models such as SHAP, PDPs, and KernalSHAP. The experimental results indicated that the AcME generates global interpretations similar to those delivered by SHAP in an element of the computation time. Furthermore, this study presented the model as a potential root-cause analysis tool intended to assist in interpreting why the algorithm classified a test as normal or abnormal; however, no structured user-centred or usability validation was conducted to confirm whether these explanations improved actual user understanding or decision-making.

### 3.6. Accumulated Local Effects (ALE)

Accumulated Local Effects (ALE) plots are another interpretability technique closely related to PDPs [68]. The aim of ALE is to address the considerable shortcoming of PDPs, which assumes independence between features. Unlike PDPs, ALE plots calculate the conditional distribution instead of the marginal distribution. To account for associated features, ALE plots compute the average differences in predictions, thus blocking the effect of associated attributes. Instead of averaging predictions, ALE averages over other features, allowing a more widespread and accurate interpretation of the model’s behaviour. From the literature, one study has been identified that used the ALE in the field of environment [44]. This study employed ALE to interpret the models’ outcomes in predicting biological stream conditions. This study also used other XAI techniques, including SHAP, PDPs, and ICE, to incorporate suggestions to peek inside black box models.

### 3.7. Explain Like I’m 5 (ELI5)

Explain Like I’m 5 (ELI5) is a Python (version 3.9) package developed to interpret black-box ML models in Python. ELI5 is capable of demonstrating the importance of each feature used by the prediction model. ELI5 determines the significance of the feature by analysing weights associated with each feature. These weights are obtained by tracing the decision paths in the tree of an assembly. This XAI technique is designed for widely used Python-based ML packages, such as XGBoost, Scikit-learn, and Keras. In the corpus of 44 studies, only one study presents the implementation of ELI5 to gain insight into solar photovoltaic power generation [7]. This study also observed that ELI5 can be demonstrated simply and contribute to the final prediction decisions across all trees and each data point. However, ELI5 does not support model-agnostic interpretations and is limited to tree-based models.

### 3.8. Explain

EXPLAIN is an XAI technique that can be applied to any ML prediction model for interpreting [69]. The key technique used in this method is sensitivity analysis, changing the inputs of the prediction model and analysing the changes in the model’s outcome [70]. This method has been utilised to explain ML models in business to business (B2B) sales predictions. This study revealed that the EXPLAIN technique could identify the most influential B2B sales features. The study also demonstrated interactive support for decision-makers evaluating various scenarios by using XAI methods. However, the EXPLAIN method is unable to capture disjunctively expressed dependencies in the prediction model. The IME method has resolved this limitation, which will be discussed in the following section.

### 3.9. IME

IME method also can be applied to any prediction model for interpreting models [71]. Like EXPLAIN, the IME method also used sensitivity analysis [70]. The application of the IME technique has been found in one article [70], which is discussed in Section 3.8. The main drawback of this method is that it may be slow on large datasets.

### 3.10. Individual Conditional Expectation (ICE)

Individual Conditional Expectation (ICE) plots were initially introduced in [72]. ICE is a model-agnostic interpretability method. This method is designed on the idea of Partial Dependence Plots (PDPs) but enhances it. The authors of the original paper identified the limitations of PDPs in capturing the complexity of relationships, mainly in scenarios where significant interaction effects exist. To overcome these limitations, the author refined the original concept and proposed a novel approach. Each plot shows the functional relationship between the predicted value and the feature for individual instances. Accordingly, a feature’s entire distribution of individual conditional expectation function is available, enabling the identification of heterogeneities and their extent. This technique has been employed by Maloney et al., (2022) [44] for interpreting predictive modelling of biological stream conditions. In ICE plots, each observation is denoted by a single line. These plots are utilised to visualise the relationships between input variables and the ML prediction outcome [44].

### 3.11. Permutation Importance (PIMP)

Permutation Importance (PIMP) is an interpretable technique that aims to correct the biased feature importance by normalising feature importance measures. The method assumes that the random significance of a feature follows a specific probability distribution and estimates its parameters by repeatedly permuting the output array of predictions and measuring the importance distribution for each variable on the non-permuted output. A recent study used PIMP to modify the biased feature importance [64] in financial distress prediction problems. In financial distress prediction, the explainability of ML models is as significant as their prediction accuracy. LR models are the most widely employed methods for evaluating feature correlations. However, LR models have biases, especially on highly noisy data, which may also lean to affect input weights, and the significance of linearly associated features can be weakened. The study demonstrated that the PIMP method indicated appropriate feature selection and was effective for most of the ML methods, such as GBDT and XGBoost.

### 3.12. KernelSHAP

KernelSHAP utilises Linear LIME [57] with Shapley values to build a Local Explanation Method. The local explanation method is a weighted linear regression, which is constructed by leveraging two components: a background set and a sample encompassing a possible coalition of features inherent in the data [73]. A recent study conducted by Dandolo et al. [56] employed KernalSHAP as a state-of-the-art model-agnostic approach to evaluate the interpretable outcomes of the ML models. The XAI techniques are applied to obtain feature importance scores both at the global and local levels. KernelSHAP provided more accurate predictions with fewer evaluations of the original model than other sampling-based predictions.

### 3.13. SHAPASH

SHAPASH is an innovative XAI tool seeking to enhance the understandability and interpretability of ML models for a broader audience [26]. This technique provides an extensive array of visually appealing interpretations with properly defined labels. The application of SHAPASH has been explored in a recent study for interpreting ML models based on time-series data for the early detection of Parkinson’s disease [26]. Their experimental work demonstrated that SHAPASH generated similar results compared to the other XAI techniques, such as SHAP and LIME.

## 4. Discussion

### 4.1. Review Summary

A total number of 44 Q1 journal articles on prediction applications in various domains where quantitative data was utilised were reviewed. Table A1 (in Appendix A) summarises the reviewed articles by extracting their attributes, including XAI techniques, AI methods, application domain, prediction problem, and data. Considering the XAI techniques utilised for predictions, 13 methods were identified. Among them, SHAP was the most popular technique as it is better known for its ability to offer accurate feature importance ranking and interpretations for individual features [20]. This aligns with recent imaging-centric studies, e.g., [10,11] in which SHAP remains among the dominant techniques used for biomedical imaging prediction tasks. The second most popular XAI technique was LIME. LIME is capable of generating explanations by approximating complex models [57]. The other two prevalent techniques after SHAP and LIME are PDPs and PFI. The PDPs method is better known for visualising the relationship between a target attribute and one or more input attributes while holding other attributes constant [64]. The PFI is known for its ability to assess the importance of features by shuffling their values. It also measures the resulting impact on model performance [66].

The XAI techniques have diverse applications in various prediction problems across numerous sectors. Based on the reviewed articles, 13 domains were identified using XAI techniques including biomedical sensing such as civil engineering, healthcare in general, energy, finance, and economics. These industries have utilised XAI techniques for various prediction tasks, such as early detection of diseases [25,26], predicting stock market prices [59], identify important factors affecting building structures [35], and predict economic growth rates [49]. The following section will discuss the challenges that need to be solved in the application of XAI techniques for predictions. Although this review spans multiple applied fields, its cross-domain comparison is essential for biomedical imaging and sensing. Understanding how explainability methods perform in other quantitative domains informs their adaptation to medical datasets, where interpretability and regulatory assurance are paramount.

There are some limitations to be acknowledged regarding this review. This study started with an extensive task of scanning over seven hundred peer-reviewed articles from two databases. As detailed in Section 2.2, a comprehensive analysis was performed on 44 included papers to summarise the recent applications of XAI in prediction tasks where quantitative databases were used. While conducting the snowballing search, it was identified that particular articles were published before 2017, outlined in the inclusion criteria. Another important consideration is that our synthesis was bounded to 2017–2023. This cut-off was intentional, as it allowed us to systematically capture the trajectory of XAI during its initial hype cycle—from the introduction of the most influential model-agnostic methods through their widespread application across domains. Although subsequent studies (2024–2025) are emerging, they often build upon, benchmark against, or refine the techniques identified in our window (e.g., SHAP, LIME, PDPs), confirming the continued relevance of our review.

Another limitation that can be considered for this review is that the formal literature search was intentionally restricted to ScienceDirect and IEEE Xplore, focusing on Q1 journal articles published between 2017 and 2023. This decision was made to facilitate a meaningful cross-domain comparison of how XAI techniques have been implemented across diverse fields, including biomedical imaging and sensing, engineering, energy, and finance, since both databases comprehensively index journals that span these domains. During the initial scoping stage, searches were also conducted in PubMed, but the team including the independent librarian observed that the vast majority of relevant biomedical AI studies indexed there linked to full-text versions hosted on ScienceDirect or IEEE Xplore. Therefore, to avoid redundancy and maintain a uniform source base, ScienceDirect and IEEE Xplore were selected as the primary databases. Nonetheless, we explicitly acknowledge this as a limitation because PubMed and other specialist biomedical databases (e.g., Web of Science Clinical Collections, PMC) may include additional domain-specific studies not captured in our dataset. Future research should incorporate these sources to provide even broader biomedical coverage.

Furthermore, the included corpus is dominated by studies employing tabular or tree-based models (e.g., Random Forest, XGBoost) paired with model-agnostic explainers such as SHAP and LIME. Deep learning architectures that are prevalent in raw medical image analysis are comparatively under-represented among Q1 journal publications during this timeframe. Consequently, the generalisability of our findings from tabular prediction contexts to end-to-end deep imaging pipelines should be regarded as provisional, warranting targeted investigation in future biomedical XAI research. We also note that none of the 44 Q1 studies included a formal human–subject usability evaluation of XAI explanations (see Table 2 and Section 4.3).

Future work can extend this survey to examine how the field evolves beyond the early hype cycle, especially in clinical imaging and sensor-based diagnostics. Recognising the impact of these articles on the implementation of XAI techniques, they could be included in the study despite not fully meeting the inclusion criteria. Furthermore, during the screening process, there were occurrences where some articles were unintentionally overlooked due to the specific keywords (explainable AI, interpretable AI, explainable artificial intelligence, explainable machine learning, interpretable machine learning) in the databases. It was observed that the index terms of certain articles did not contain the aforementioned search keywords despite the fact that the interchangeable usage of several closely related terms within the articles’ bodies (e.g., explainability, interpretability, and whitening black box) and XAI model names (e.g., SHAP and LIME) in metadata hinders the proper acquisition of knowledge on XAI techniques. As a result, a few studies could have been missed during the review process. The absence of knowledge obtained from these omitted articles can be considered as limitation of this systematic review.

### 4.2. Challenges in Existing XAI Application and Future Direction

XAI techniques play an important role in prediction applications by providing explanations and interpretations of the predictions made by AI models [7,8]. XAI techniques enable users to make informed decisions, build trust in the prediction models, and detect biases. Reflecting a critical gap, there are a few domains currently exist frequently in applying XAI techniques for predictions apart from biomedical imaging and sensing, such as engineering, energy, and finance. These applications involved various prediction problems, comprising disease diagnosis, structural responses, load prediction, and stock market predictions. Generally, insights into model predictions are provided by XAI methods, which also analyse the impact of changing features and highlight the significant input attributes that influence prediction outcomes. These techniques aid in obtaining interpretability, comprehending the reasoning behind predictions, and making informed decisions. These existing applications demonstrate the potential of XAI in improving interpretability and trust in AI models, which can be highly beneficial in the domain on medical imaging and sensing. In biomedical imaging and sensing, XAI offers additional advantages that go beyond general interpretability. For example, in magnetic-resonance and ultrasound imaging (MRI), quantitative descriptors such as texture, intensity, or signal-to-noise ratios (SNRs) can be mapped to physiological or pathological conditions. Explanations derived from XAI allow clinicians to link algorithmic predictions to known biomarkers or sensor-derived measurements, thereby strengthening diagnostic confidence [49,68]. However, this translation from numerical importance values to clinically meaningful reasoning introduces domain-specific difficulties related to data heterogeneity, annotation quality, and interpretive validity [23]. The application of XAI techniques for predictions poses various challenges and gaps that require to be addressed to improve the effectiveness and trustworthiness of the methods.

One of these challenges is the identification of variable interactions [51,70]. In prediction problems, it is essential to understand how different input variables interact with each other and contribute to the overall forecast result. Modifying existing XAI techniques to identify relevant variable interactions or visualise the combined effect of variables is important to enhance the models’ effectiveness for decision-making. Therefore, there is a need for further investigation and the development of existing methods or new methods that can effectively capture and visualise this combined effect which can help to build trust, especially for biomedical experts in healthcare domain. For example, in multimodal sensing environments, such as combining electroencephalography (EEG) with functional near-infrared spectroscopy (fNIRS)—interactions between modalities may reveal latent physiological dependencies that one-variable-at-a-time explanations cannot capture [75]. Extending XAI techniques to visualise these cross-sensor interactions would be particularly valuable for personalised diagnostics and adaptive monitoring systems.

Another challenge is the transparency and understanding of complex neural networks, such as deep learning neural networks [50]. Deep learning models are powerful tools for solving complex prediction problems. However, the experiment lies in the inadequate availability of explanations for such methods in predictions. Developing robust and interpretable techniques for explaining deep learning models is necessary to enhance transparency. The challenge is compounded by the very high dimensionality of image or volumetric data, where a single model decision may depend on thousands of correlated pixel-level features. This opacity becomes particularly problematic in biomedical imaging tasks such as radiology or histopathology, where clinicians require visual or feature-level justification of predictions. Recent surveys (e.g., [10]) emphasise that without interpretable outputs, clinical adoption is significantly hampered.

Particular areas require further research and advancements in applying XAI for predictions. One such area is the application of XAI techniques on time series databases in the business context. Currently, limited studies have incorporated XAI on time series data, comprising predicting stock market price [59] and understand control capacity of building energy [38]. The main purpose of these studies of incorporating with XAI is to understand trends, seasonality, and other temporal patterns in the data. Nevertheless, there is a lack of knowledge in the application of XAI techniques on time series databases, which encompass different dimensions. When making business decisions, the predictions can be made based on three dimensions, comprising time, product, and supply chain. The time dimension signifies the granularities of data. For example, hourly and daily [76]. The product category in the context of time series data, which comprises brand, category, and SKU (stock keeping unit) levels [76]. The supply chain or hierarchy in the time series context refers to the structural arrangement of the data. In such scenarios, XAI techniques may be required to account for these factors and provide explainable insights at different levels, enabling better decision-making across businesses. Similarly, for biomedical sensing applications, this generalisability issue is amplified by patient heterogeneity and device variability. Furthermore, calibration drift and signal artefacts introduced by different acquisition hardware can lead to inconsistent feature rankings across patient cohorts. Consequently, explainability methods need calibration-aware adaptations that distinguish between physiological variability and device-induced discrepancies. For example, wearable sensor outputs differ across manufacturers and patient populations, which complicates the direct transfer of explanation models. Further research is required to develop or apply XAI techniques that can interpret these factors or combinations of features that have the most significant impact on prediction outcomes.

Furthermore, the evaluation and validation of XAI techniques require further research attention. Despite the existence of various XAI techniques, their comparative evaluation and validation are lacking. Establishing benchmark datasets and error metrics specific to the prediction problems would help to evaluate the performance and reliability of XAI methods [77]. The benchmark datasets should encompass a wide range of real-world scenarios, ensuring that XAI methods can be thoroughly tested and validated against each other. For example, the domains such as soil liquefaction potential assessment or biomass torrefaction have unique attributes and data constraints [40]. Addressing this domain-specific challenge is essential to improve the performance, adaptability, and evaluation of XAI methods [78]. In medical imaging, this challenge is even more acute because the usefulness of explanations is judged not only by quantitative fidelity but also by clinical interpretability. Evaluating explanations therefore requires multi-level validation that integrates quantitative metrics (e.g., fidelity, stability) with qualitative human-centred assessments such as clinician surveys or reader-study agreement scores [74]. A recent review in mammography highlights that standard metrics often fail to capture whether explanations truly aid radiologists’ decision-making [79]. Incorporating such hybrid evaluation protocols will enable researchers to quantify not only how accurately an explanation reflects model logic, but also how well it aligns with human diagnostic reasoning [80].

Finally, future research should focus on incorporating domain experts’ insights into XAI techniques, which would improve the usefulness and effectiveness of the interpretations provided. Integrating XAI with causal reasoning would accelerate the understanding of cause–effect relationships and help to discover actionable insights into prediction models’ outcomes. By implementing these approaches, stakeholders can gain a better understanding of AI systems, enabling them to make informed decisions based on transparency and trustworthy insights. The findings also encourage stakeholders from other disciplines, such as retail businesses, where XAI is not widely utilised, to understand and appreciate the merits of XAI techniques to enhance the decision-making process [81]. Stakeholders from other disciplines, particularly in biomedical imaging and sensing, should embrace the merits of XAI in their domains without preconceived notions regarding its lack of interpretability. Given the life-critical consequences in medical imaging and sensor-based diagnostic systems, the transparency afforded by XAI is not optional but essential for trust, regulatory approval, and patient safety.

Collectively, these challenges demonstrate that while XAI principles are general, their successful implementation in biomedical contexts depends on domain-specific data curation, clinically meaningful validation, and integration with existing diagnostic workflows. Looking forward, biomedical imaging and sensing represent frontier domains where XAI integration is both most urgent and most challenging [22]. Clinical workflows demand explanations that are fast, intuitive, and robust under real-world conditions, yet current post hoc methods (e.g., SHAP, LIME) can be computationally expensive and difficult for non-experts to interpret. Future work should focus on developing domain-tailored explanation frameworks co-designed with clinicians and biomedical engineers, alongside standardised protocols for evaluating clinical utility of explanations. Such advances will be essential for regulatory approval and widespread adoption of AI-powered sensing technologies.

This cross-domain synthesis also highlights clear translational gaps for biomedical imaging and sensing: most techniques validated in engineering or finance are yet to be adapted to clinical data with real-user validation. In addition, it is important to recognise theoretical and practical critiques of widely used explainers (notably SHAP) that have direct implications for biomedical deployment. The growing literature (e.g., [22,23,24,75] questions whether Shapley-value based explanations always provide faithful or causally meaningful interpretations in real data settings. Specific concerns include sensitivity to feature correlation, dependence on the chosen background distribution for marginalisation [82], and limits to causal interpretation when applied to heterogeneous biomedical attributes (e.g., temperature, blood pressure, biomarkers) that are not homogeneous payoff units as in classical game theory [22]. As SHAP and similar additive attributions assume decomposability of contributions, their outputs can be misleading if those assumptions do not hold; therefore, biomedical researchers must apply such methods cautiously, consider conditional or causal formulations where appropriate, and combine computational attributions with domain validation (e.g., clinician review, controlled user studies) before inferring clinical meaning [23]. The methodological comparison, presented in this study is therefore directly informative for biomedical researchers seeking to select or modify XAI methods suitable for regulated, safety-critical environments.

### 4.3. Usability, and Clinical Utility of XAI

This study highlights a key gap in the literature about the limited evidence of XAI methods actually increasing the end-user confidence or improve decision outcomes in practice. The studies included in this review mostly generate post hoc explanations (i.e., feature importance, PDPs, SHAP values, LIME) and do not evaluate whether those explanations are usable, actionable, or trusted by domain experts. A minority of studies explicitly report human-in-the-loop evaluations (for example Dandolo et al. [56] discussed human-in-the-loop decision support), while broader empirical work (e.g., Lahav et al. [81]) and recent critical studies [22,23,24,75] demonstrate that some XAI methods may fail usability tests or be misleading in practice.

Among studies included in this review, none reported a formal human–subject usability study assessing whether XAI explanations improve clinician/user trust or decision-making. The studies presented in Table 2 are representative examples showing that evaluation was limited to computational analyses, retrospective clinical validation of model predictions, or expert commentary.

The literature indicates three important distinctions that should be considered when assessing clinical utility: (i) Generation vs. validation, (ii) Fidelity vs. usefulness, (iii) Workflow fit. For example, a SHAP bar plot is not equivalent to validating that clinicians find it meaningful or that it improves decisions [22]. Similarly, methods with high computational fidelity may produce outputs that are not clinically interpretable [75]; conversely, simpler explanations may be more useful to clinicians [24]. Typically, clinical adoption depends on how explanation outputs integrate with existing workflows (timeliness, format, and regulatory documentation) [82]. The representative examples in Table 2 illustrate a recurring pattern across the studies included in this review, reporting computational assessments of XAI outputs (feature importance rankings, fidelity/stability comparisons, runtime/approximation efficiency) and in some cases clinical validation of model predictions (retrospective or prospective performance), but none of the included studies conducted a structured human–subject usability experiment that measures whether XAI explanations improve clinician or end-user decision performance, trust, or acceptability. Therefore, while many studies generate explanations, empirical evidence that these explanations deliver measurable user benefit in biomedical imaging or sensing workflows remains absent in the selected corpus.

We therefore recommend that future work explicitly tag included studies on whether they performed user evaluations, simulated clinical tasks, or workflow integration experiments, and we encourage researchers to adopt standardised human-evaluation protocols (usability testing, task performance, clinician survey instruments). Until such evidence accumulates, claims that a method is “effective” should be qualified as “effective for generating post hoc explanations” rather than “effective at improving clinical decisions.”

### 4.4. Relevance of XAI to Biomedical Imaging and Sensing

Although this review covered a broad spectrum of quantitative prediction applications, one of the most critical gaps has been the application of eXplainable XAI is biomedical imaging and sensing. The adoption of AI in clinical environments depends not only on accuracy but also on transparency, since clinicians, patients, and regulators must understand how model predictions are derived [23]. Medical imaging modalities (e.g., CT, MRI, mammography) and sensor-based monitoring systems (e.g., wearable devices, biosensors) produce large-scale quantitative datasets where black-box models pose significant risks if left uninterpreted.

Recent surveys underscore this growing emphasis. For example, works published in 2025 about the use of XAI for medical imaging systems via deep learning [10,11,81], which provides a state-of-the-art synthesis of XAI in imaging, highlighting both post hoc explanation methods such as SHAP and Grad-CAM, and self-explainable approaches tailored for clinical workflows. Similarly, self-eXplainable AI for medical image analysis [11,22] published in 2024, which stresses the importance of designing models with built-in interpretability to improve clinician trust and usability. In specific modalities, a review of explainable AI techniques and their evaluation in mammography [79], published in 2025, shows how explanation quality can directly influence radiologist decision support, while the use of XAI in medicine [22] also published in 2025, discusses barriers to regulatory and clinical adoption.

Collectively, these recent works confirm the timeliness of situating our review within the biomedical context. Although our inclusion window (2017–2023) was deliberately defined to capture the first hype cycle of XAI methods, the techniques identified—particularly SHAP, LIME, and PDPs—remain the same tools that biomedical researchers continue to adopt and evaluate in 2024–2025. This indicates that our systematic synthesis provides a relevant foundation for advancing trustworthy AI in biomedical imaging and sensing.

Future updates to this review may expand to formally include the rapidly growing body of biomedical-focused studies, but even within our dataset, healthcare applications illustrate how interpretable prediction methods can bridge the gap between technical performance and clinical trust. As biomedical sensing technologies proliferate, the lessons drawn from this cross-domain review remain critical for ensuring that AI-driven diagnostic and monitoring systems are safe, reliable, and explainable.

## 5. Conclusions

This study systematically reviews XAI techniques’ contribution to prediction applications where quantitative data was utilised. As a result, 44 Q1 journal articles were suitable for inclusion in this review. Considering the growing interest in XAI, it is significant to have a comprehensive overview of the application of XAI for predictions through this review. This review facilitates the application of XAI techniques by illustrating a collection of the most appropriate works and providing clear descriptions of the XAI techniques and their applications in various domains.

The studies included in our systematic review were categorised based on the respective XAI techniques and application domains. Among the identified models, SHAP was the most frequently applied technique for computationally interpreting AI models in prediction tasks. SHAP is often used in conjunction with XGBoost and RF, reflecting its compatibility with models that offer quantitatively strong predictive performance and post hoc explainability. Another popular XAI technique for prediction applications is LIME, which offers visual explanations by emphasising essential features that influence prediction outcomes.

In addition, this study highlighted the limited number of XAI studies focusing on interpreting prediction outcomes in time series forecasting where the AI models are extensively applied. These areas would demand attention for future research on evaluating XAI techniques for interpreting AI models. By focusing on 2017–2023, our review captures the first major hype cycle of XAI development, highlighting the techniques that established themselves as benchmarks in interpretable prediction. These insights provide a stable foundation upon which newer biomedical imaging and sensing applications (emerging post-2023) continue to build. Overall, this review provides not only a cross-domain synthesis of quantitative XAI implementations but also a translational lens for biomedical imaging and sensing. By identifying the methodological and usability gaps that must be bridged for clinical deployment, this study provides guidance for adapting existing explainability frameworks to safety-critical biomedical contexts. Future research should aim to update the synthesis to include more recent publications, especially those in biomedical imaging and sensing, and to establish standardised reporting guidelines for prediction tasks (e.g., how feature importance is evaluated, data used, metrics). Additionally, the field would benefit from longitudinal studies tracking how well explainability methods are adopted in clinical imaging or sensor-based diagnostic devices.

## Figures and Tables

**Table 1:** Search strategy for the selected literature.

Database	Article Parts Searched	Field	Search String
ScienceDirect	Title, Abstract	All Fields	((“Explainable AI” OR “interpretable AI” OR “explainable artificial intelligence” OR “interpretable artificial intelligence” OR “explainable machine learning” OR “interpretable machine learning”) AND (“Biomedical Imaging” OR “Biomedical Sensing” OR “Biomedical” OR “Imaging” OR “Sensing”))
IEEE	Title, Abstract	All Fields	((“All Metadata”: explainable AI) OR (“All Metadata”: interpretable AI) OR (“All Metadata”: explainable machine learning) OR (“All Metadata”: interpretable machine learning) OR (“All Metadata”: interpretable machine learning) AND (“Biomedical Imaging” OR “Biomedical Sensing” OR “Biomedical” OR “Imaging” OR “Sensing”))

**Table 2:** Formal human–subject usability evaluation of XAI explanations.

Studies	Domain/Application	XAI Method Used	Primary Evaluation Reported	Formal Usability
[1]	Healthcare COVID-19 diagnosis	LIME, SHAP	Model predictive performance (classification metrics) + computational explanations (feature importance/visuals)	No (presents computational evaluation of predictability)
[25]	Healthcare—prognostic variables	SHAP, PDPs, PFI	Feature importance analyses; model performance metrics	No (presents feature extraction for clinical validity)
[26]	Miscellaneous/human-in-the-loop example	SHAP, LIME, SHAPASH	Computational explanation comparisons; predictive accuracy	No (presents data experimentation)
[28]	Healthcare—prognostic variables	SHAP, LIME, PFI	Insights into prognostic variables; model performance; and clinical data analysis	No (presents clinical validation of prediction)
[29]	Seminal/Method paper (LIME)	LIME	Method demonstration and computational examples	No (presents method proposal/computational evaluation)
[56]	Miscellaneous/human-in-the-loop example	SHAP, AcME, KernelSHAP, PDPs	Computational runtime/efficiency and fidelity comparisons; demonstration as root-cause tool	No (demonstrates potential for human-in-loop; no usability trials)
[74]	Healthcare—disease detection	SHAP,	Prediction performance on ECG/retrospective clinical dataset; feature importance	No (presents EEG Data Evaluations with Experimental results

## Data Availability

The original contributions presented in this study are included in the article/Supplementary Material. Further inquiries can be directed to the corresponding authors.

## Supplementary Materials

The following supporting information can be downloaded at: https://www.mdpi.com/article/10.3390/s25216649/s1, PRISMA 2020 Checklist.

## Appendix A

**Table A1:** Forty-four Q1 journal articles incorporating XAI techniques.

Authors	Year	XAI Method	Classifier	Industry	Application	Data
[1]	2023	LIME, SHAP	XGBoost	Healthcare	Diagnosis of COVID-19	COVID-19 positive and negative patients
[59]	2023	LIME	RF	Finance	Prediction of stock market prices.	Stock market price
[42]	2023	LIME, SHAP	ET, XGBoost	Environmental	Prediction of daily pan evaporation.	Air temperature (Ta), solar radiation (Rs), and relative humidity (H)
[56]	2023	SHAP, AcME, KernalSHAP, PDPs	XGBoost	Miscellaneous	Predict abnormal behaviours, assessing the impact of changes in feature values on model predictions, and identifying feature importance and ranking.	Chemical feature Glass Data
[45]	2023	PDPs, SHAP	GBR	Finance	Predict daily stock prices.	Stock market prices
[55]	2023	SHAP	Poly, RFR, XGBoost, DNN	Mining	Predict the ash content of coal samples based on the composition data of XRF analysis.	XRF data
[43]	2023	SHAP	XGBoost	Environmental	Predict the responsibility of environmental factors in changing water. quality from eutrophic to hypereutrophic states.	Water quality
[47]	2023	SHAP	RF	Chemical Engineering	Predict the CO_2_ adsorption capacity.	CO_2_ adsorption capacity of PC based on TP
[37]	2023	SHAP	NGB	Energy	Assess the relationship between hydrogeochemical variables and reservoir temperature.	predict reservoir temperature u
[30]	2023	SHAP	XGBoost	Civil Engineering	Identify important input parameters affecting the liquefaction potential.	Historical post-liquefaction
[25]	2023	SHAP, PDPs, PFI	XGBoost	Healthcare	Early diagnosis of chronic kidney disease (CKD).	Haemoglobin, specific gravity, and hypertension
[31]	2023	SHAP	ANN	Civil Engineering	Probabilistic buckling stress prediction models of steel shear panel dampers.	Steel shear panel damper
[32]	2023	SHAP	RF	Civil Engineering	Predict seismic drifts in CLT buildings.	Drift demands
[53]	2023	SHAP	SVR	Genomics	Predicting the martensitic transformation peak temperature and investigating the effects of important features and alloying elements on TP for TiZrHfNiCoCu HESMAs.	Temperature
[26]	2023	SHAP, LIME, SHAPASH	LGBM	Healthcare	Prediction of Parkinson’s Disease (PD) progression.	Clinical data
[49]	2022	SHAP	LSTM	Economic	Predict economic growth rates and crises by capturing sequential dependencies within the economic cycle.	GDP growth rates
[41]	2022	SHAP, LIME	LSTM	Energy	Building load prediction.	Energy consumption data
[60]	2022	LIME	LSTM	Mechanical Engineering	Predict temperature in the District Heating Systems’ (DHS) supply line.	DHS operation
[51]	2022	SHAP	LR	Aeronautics	Predicting the failure of components and systems in jet engines.	Run-to-failure trajectories for jet engines
[52]	2022	SHAP	XGBoost	Aviation	Predict demand of air transportation.	Weather, runway reports, flight data
[38]	2022	SHAP	GBTs	Energy	Analyse secondary control activation.	FRR exhibit fixed time scales and deadlines
[61]	2022	LIME	RF	Sports	Analyse the match style and gameplay of the national basketball association (NBA).	NBA gameplay data at a seasonal level
[77]	2022	SHAP	XGBoost	Healthcare	Predict the malnutrition status of children with CHD using explainable ML methods to provide insight into the model’s predictions and outcomes.	Cohort data
[33]	2022	SHAP	XGBoost	Civil Engineering	Predicting the shear capacity of FRP-RC beams. n (SHAP) is used to identify the most important factors that influence the shear capacity prediction of FRP-RC beams.	Shear critical FRP-RC beams
[39]	2022	SHAP		Energy	Power factor prediction model with high accuracy and introduced interpretability tools to gain material-oriented insight.	Power factors
[44]	2022	PDPs, ALE, ICE, SHAP	RF	Environmental	Predict biological stream conditions in the Chesapeake Bay Watershed, USA.	Catchments
[27]	2022	SHAP	MLP	Healthcare	Prediction of post-operative mortality after any surgery in an emergency setting on elderly patients.	Demographic and clinical data, medical and surgical history, preoperative risk factors, frailty, biochemical blood examination, vital parameters, and operative details
[46]	2022	SHAP	XGBoost	Finance	Predict financial pressure.	financial pressure and the volatility of the healthcare stock market
[34]	2022	SHAP	XGBoost	Civil Engineering	Identify failure mode of RC flat slabs without transverse reinforcement.	610 groups of data
[35]	2022	SHAP	XGBoost	Civil Engineering	Predict external wind pressure of a low-rise building in urban-like settings.	Eexternal wind pressure
[48]	2022	SHAP	XGBoost	Chemical Engineering	Predict external wind pressure of a low-rise building in urban-like settings.	Azo molecules
[40]	2022	SHAP	GTB	Energy	Predict yields and higher heating value of torrefied biomass.	Torrefaction data
[29]	2022	SHAP	XGBoost	Healthcare	Understand the main logic governing the model prediction.	Blood count
[36]	2022	SHAP	XGBoost	Civil Engineering	Prediction of one-part alkali-activated material enabled by interpretable machine learning.	Alkali activated material (AAM)
[66]	2021	PFI	SVM	Civil Engineering	Analysing the mechanical performance of fly ash-based geopolymer concrete with different machine learning techniques.	Fly ash (FA)-based geopolymer concrete, hydroxide (NaOH) and sodium silicate (Na_2_SiO_3_)
[28]	2022	SHAP, LIME, PFI	XGBoost	Healthcare	Understand previously undetected relationships between prognostic variables to make informed clinical decisions and effective interventions.	Clinical data
[64]	2021	PIMP, PDPs	XGBoost	Finance	Predict financial distress	Accounting records and financial ratios
[78]	2021	SHAP	RF	Economic	Evaluate the precision of housing-price forecasts.	Housing Prices
[65]	2021	PDPs	RF	Environmental	Predict hydrogen production.	Hydrogen production
[74]	2020	SHAP	XGBoost	Healthcare	The early and accurate detection of the onset of acute myocardial infarction (AMI).	Electrocardiogram Vigilance with Electronic data
[7]	2020	LIME, SHAP, ELI5	RF	Energy	Gaining insight into solar photovoltaic power generation forecasting.	Weather
[67]	2020	PFI	RF	Civil Engineering	Predict structural response of RC slabs exposed to blast loading.	Reinforced concrete slabs exposed to blast loading using ten (input)
[62]	2019	LIME		Civil Engineering	Explain and evaluate data-driven building energy performance.	Building and BAS data
[70]	2017	EXPLAIN, IME	RF	Finance	Business-to-business (B2B) sales forecasting.	B2B sales data
