# Supplementary material for: eXplainable Artificial Intelligence (XAI): A Systematic Review for Unveiling the Black Box Models and Their Relevance to Biomedical Imaging and Sensing
Source: Sensors (Basel). 2025 Oct 30;25(21):6649. doi: 10.3390/s25216649 (PMC12609895; doi:10.3390/s25216649)
Supplement: Supplementary file 1 [file sensors-25-06649-s001.zip › sensors-3926491-supplementary.pdf]

PRISMA 2020 Checklist

Section and Topic	Item #	Checklist item	Location where item is reported
**TITLE**			
Title	1	Identify the report as a systematic review.	Yes, reported in Title page.
**ABSTRACT**			
Abstract	2	See the PRISMA 2020 for Abstracts checklist.	Yes, structured abstract provided (see Abstract section).
**INTRODUCTION**			
Rationale	3	Describe the rationale for the review in the context of existing knowledge.	Introduction, first and second paragraphs.
Objectives	4	Provide an explicit statement of the objective(s) or question(s) the review addresses.	Introduction, end of section (explicit aim/objectives stated).
**METHODS**			
Eligibility criteria	5	Specify the inclusion and exclusion criteria for the review and how studies were grouped for the syntheses.	Section 2.2. Selection Criteria and Quality Assessment (Eligibility / inclusion-exclusion described; see Fig. 1 and Appendix Table II
Information sources	6	Specify all databases, registers, websites, organisations, reference lists and other sources searched or consulted to identify studies. Specify the date when each source was last searched or consulted.	Section 2.1. Search Strategy and Table I (ScienceDirect, IEEE; date range: 2017 to March 2023 with justification)
Search strategy	7	Present the full search strategies for all databases, registers and websites, including any filters and limits used.	Section 2.1. Search Strategy and Table I (full search strings provided in Table I).
Selection process	8	Specify the methods used to decide whether a study met the inclusion criteria of the review, including how many reviewers screened each record and each report retrieved, whether they worked independently, and if applicable, details of automation tools used in the process.	Section 2.2. Selection Criteria and Quality Assessment and Figure 1 (PRISMA filtration flow); screening steps and duplicate removal described.
Data collection process	9	Specify the methods used to collect data from reports, including how many reviewers collected data from each report, whether they worked independently, any processes for obtaining or confirming data from study investigators, and if applicable, details of automation tools used in the process.	Section 2.3. Data Extraction and Analysis (procedures for extracting study attributes and how data were organised).
Data items	10a	List and define all outcomes for which data were sought. Specify whether all results that were compatible with each outcome domain in each study were sought (e.g. for all measures, time points, analyses), and if not, the methods used to decide which results to collect.	Section 2.3. Data Extraction and Analysis (RQ and attributes; outcomes/metrics

Section and Topic	Item #	Checklist item	Location where item is reported
			sought described in the attributes list).
	10b	List and define all other variables for which data were sought (e.g. participant and intervention characteristics, funding sources). Describe any assumptions made about any missing or unclear information.	Section 2.3. Data Extraction and Analysis (variables: XAI methods, prediction model, application domain, data types; see Appendix Table II).
Study risk of bias assessment	11	Specify the methods used to assess risk of bias in the included studies, including details of the tool(s) used, how many reviewers assessed each study and whether they worked independently, and if applicable, details of automation tools used in the process.	Section 2.2. A formal risk-of-bias assessment tool was not applied, as the review focused on mapping techniques rather than clinical effect estimates. However, to reduce bias, an independent reviewer (librarian) was involved in the screening and data extraction process.”
Effect measures	12	Specify for each outcome the effect measure(s) (e.g. risk ratio, mean difference) used in the synthesis or presentation of results.	Not applicable (no meta-analysis; no effect measure pooled).
Synthesis methods	13a	Describe the processes used to decide which studies were eligible for each synthesis (e.g. tabulating the study intervention characteristics and comparing against the planned groups for each synthesis (item #5)).	Section 2.3. Data Extraction and Analysis (description of how included studies were grouped and the attributes used to organise syntheses).
	13b	Describe any methods required to prepare the data for presentation or synthesis, such as handling of missing summary statistics, or data conversions.	Section 2.3. Data Extraction and Analysis (data organisation, handling of overlapping/multiple techniques, and tabulation described).
	13c	Describe any methods used to tabulate or visually display results of individual studies and syntheses.	Section 3. Results; Figures 2–5; Appendix Table II (methods used to tabulate and display results).
	13d	Describe any methods used to synthesize results and provide a rationale for the choice(s). If meta-analysis was performed, describe the model(s), method(s) to identify the presence and extent of statistical heterogeneity, and software package(s) used.	Not applicable, as no meta-analysis was conducted.
	13e	Describe any methods used to explore possible causes of heterogeneity among study results (e.g. subgroup analysis, meta-regression).	Not applicable (no statistical heterogeneity analysis; narrative cross-domain comparisons in Results and

Section and Topic	Item #	Checklist item	Location where item is reported
			Discussion).
	13f	Describe any sensitivity analyses conducted to assess robustness of the synthesized results.	Not applicable, as no meta-analysis was conducted.
Reporting bias assessment	14	Describe any methods used to assess risk of bias due to missing results in a synthesis (arising from reporting biases).	Formal assessment of reporting bias (e.g., funnel plots, Egger's test) was not applicable, as no quantitative meta-analysis of effect estimates was performed. However, we attempted to mitigate reporting bias by systematically searching multiple databases, screening reference lists, and including only Q1 journal publications to ensure high-quality peer-reviewed evidence. Grey literature and Google Scholar were also checked to confirm no state-of-the-art review articles were missed.
Certainty assessment	15	Describe any methods used to assess certainty (or confidence) in the body of evidence for an outcome.	Not applicable (no formal certainty assessment performed). Rationale and mitigations described in Section 2.2. Selection Criteria and Quality Assessment and in Discussion (4.1 Review Summary / Limitations). Since this review focused on mapping and characterising XAI techniques rather than evaluating clinical intervention outcomes. Instead, certainty was indirectly addressed by restricting the dataset to Q1 journal publications and cross-validating extracted information through independent review to

PRISMA

PRISMA 2020 Checklist

Section and Topic	Item #	Checklist item	Location where item is reported
			ensure accuracy.
**RESULTS**			
Study selection	16a	Describe the results of the search and selection process, from the number of records identified in the search to the number of studies included in the review, ideally using a flow diagram.	Section 2.2. Selection Criteria and Quality Assessment; Figure 1 (PRISMA 2020 systematic review filtration protocol showing records identified, screened, excluded, included)
	16b	Cite studies that might appear to meet the inclusion criteria, but which were excluded, and explain why they were excluded.	Section 2.2. Selection Criteria and Quality Assessment and also the Figure 1 (reasons for exclusion summarised).
Study characteristics	17	Cite each included study and present its characteristics.	Appendix Table II (“44 Q1 Journal Articles incorporating XAI Techniques”) and Section 3 Results (and subsections 3.1–3.13).
Risk of bias in studies	18	Present assessments of risk of bias for each included study.	Not applicable (no formal RoB assessment was performed). Mitigation steps and rationale are reported in Section 2.2. Selection Criteria and Quality Assessment.
Results of individual studies	19	For all outcomes, present, for each study: (a) summary statistics for each group (where appropriate) and (b) an effect estimate and its precision (e.g. confidence/credible interval), ideally using structured tables or plots.	Sections 3.1–3.13 (individual XAI technique subsections) and Appendix Table II (per-study attributes and data).
Results of syntheses	20a	For each synthesis, briefly summarise the characteristics and risk of bias among contributing studies.	Section 4.1. Review Summary and Appendix Table II (summary of characteristics); note: RoB assessment not performed (see Section 2.2 bias mitigation).
	20b	Present results of all statistical syntheses conducted. If meta-analysis was done, present for each the summary estimate and its precision (e.g. confidence/credible interval) and measures of statistical heterogeneity. If comparing groups, describe the direction of the effect.	Not applicable (no meta-analysis).
	20c	Present results of all investigations of possible causes of heterogeneity among study results.	Not applicable (no statistical

Section and Topic	Item #	Checklist item	Location where item is reported
			heterogeneity analyses). Narrative discussion of cross-domain differences in Results and Discussion (B. Challenges in Existing XAI Application and Future Direction).
	20d	Present results of all sensitivity analyses conducted to assess the robustness of the synthesized results.	Not applicable.
Reporting biases	21	Present assessments of risk of bias due to missing results (arising from reporting biases) for each synthesis assessed.	Not applicable (no statistical synthesis). Mitigation and discussion in Section 2.1. Search Strategy; Section 2.2. Selection Criteria and Quality Assessment; Discussion (Section 4.1 Review Summary / Limitations).
Certainty of evidence	22	Present assessments of certainty (or confidence) in the body of evidence for each outcome assessed.	Not applicable (no formal assessment). Rationale and mitigation described in Section 2.2 and Discussion (Section 4.1).
**DISCUSSION**			
Discussion	23a	Provide a general interpretation of the results in the context of other evidence.	Section 4.1. Review Summary.
	23b	Discuss any limitations of the evidence included in the review.	Section 4.1. Review Summary (Limitations paragraphs).
	23c	Discuss any limitations of the review processes used.	Section 4.1. Review Summary (Limitations paragraphs; includes 2017–2023 cut-off rationale, keyword/indexing limitations, and Q1 restriction).
	23d	Discuss implications of the results for practice, policy, and future research.	Section 4.1. B. Challenges in Existing XAI Application and Future Direction and C. Relevance of XAI to Biomedical Imaging and Sensing.
**OTHER INFORMATION**			
Registration and	24a	Provide registration information for the review, including register name and registration number, or state that the review was not	Section 2.1. Search Strategy: "The review

PRISMA 2020 Checklist

Section and Topic	Item #	Checklist item	Location where item is reported
protocol		registered.	protocol was not prospectively registered in PROSPERO or other registries." (statement included).
	24b	Indicate where the review protocol can be accessed, or state that a protocol was not prepared.	Section 2.1. Search Strategy: No protocol prepared (state provided).
	24c	Describe and explain any amendments to information provided at registration or in the protocol.	Not applicable.
Support	25	Describe sources of financial or non-financial support for the review, and the role of the funders or sponsors in the review.	Acknowledgements/Funding section
Competing interests	26	Declare any competing interests of review authors.	Conflict of Interest statement at the end of the manuscript
Availability of data, code and other materials	27	Report which of the following are publicly available and where they can be found: template data collection forms; data extracted from included studies; data used for all analyses; analytic code; any other materials used in the review.	Methods/Availability Statement at the end of the manuscript.

From: Page MJ, McKenzie JE, Bossuyt PM, Boutron I, Hoffmann TC, Mulrow CD, et al. The PRISMA 2020 statement: an updated guideline for reporting systematic reviews. BMJ 2021;372:n71. doi: 10.1136/bmj.n71. This work is licensed under CC BY 4.0. To view a copy of this license, visit https://creativecommons.org/licenses/by/4.0/
